# Evaluation of Activity of Pro- and Anti-Inflammatory Mediators and Nitrosative Stress in Liver Tissue of Wild Boars (*Sus scrofa*) Positive for Zearalenone (ZEN) Contamination in Campania Region, Southern Italy

**DOI:** 10.3390/toxins17110553

**Published:** 2025-11-05

**Authors:** Sara Damiano, Consiglia Longobardi, Evaristo Di Napoli, Valentina Iovane, Francesco Ferrucci, Giuseppe Rizzo, Antonio Raffaele, Antonio Rubino, Valeria Russo, Roberto Ciarcia

**Affiliations:** 1 Department of Veterinary Medicine and Animal Production, University of Naples “Federico II”, 80137 Naples, Italy; sara.damiano@unina.it (S.D.); francescoferrucci92@icloud.com (F.F.); antoruby@virgilio.it (A.R.); valeria.russo@unina.it (V.R.); rciarcia@unina.it (R.C.); 2 Department of Agricultural Sciences, University of Naples Federico II, 80055 Portici, Italy; valentina.iovane@unina.it; 3 Component of the Local Management Hunting Authority (ATC), Province of Avellino, Campania Region, 83100 Avellino, Italy; giusepperizzo886@gmail.com; 4 President of the Local Management Hunting Authority (ATC), Province of Avellino, Campania Region, 83100 Avellino, Italy; raffaeleantonio@libero.it

**Keywords:** zearalenone, wild boar, liver, nitrosative stress, inflammation

## Abstract

Zearalenone (ZEN) is a mycotoxin commonly produced by *Fusarium* species and is often found in food and feed. It has been linked to reproductive problems in livestock and, less frequently, to hyperestrogenic effects in humans. However, information regarding the impact of ZEN on wild boars (*Sus scrofa*) remains scarce, despite this species being among the most frequently hunted game animals in Italy. The aim of this study was to assess the impact of ZEN on the hepatic system by examining nitrosative stress markers and the balance between pro- and anti-inflammatory cytokines in wild boars hunted in various areas of the Avellino province (Campania region, Italy) during the 2021–2022 hunting season. The findings revealed that exposure to ZEN was linked to a marked rise in both pro- and anti-inflammatory mediators, except for IL-10, which did not increase significantly. In addition, ZEN stimulated the expression of inducible nitric oxide synthase (iNOS), which, in turn, led to elevated nitric oxide (NO) concentrations in the liver. The immunohistochemical analysis revealed a predominance of CD3-positive T-cells in the hepatic inflammatory infiltrate of ZEN-exposed wild boars, highlighting the importance of structured wildlife monitoring to protect food safety and safeguard human and animal health.

## 1. Introduction

Zearalenone (ZEN) is a common food contaminant and a non-steroidal estrogenic mycotoxin primarily produced by fungi of the *Fusarium* species. This low-molecular-weight compound is frequently found in different crops and geographic areas because of its remarkable thermal stability and persistence [1]. Due to these characteristics, ZEN tends to build up in significant amounts throughout various stages of food production, including harvesting, storage, and processing, posing a significant concern for both animal and human health [2,3]. The problem is exacerbated by the climate, the most important factor affecting fungal colonization and mycotoxin production [1,4]. At present, ZEN contamination is worldwide, with the highest concentrations reported in temperate and warmer climate regions, including Europe, America and Asia [4]. A wide variety of cereals such as barley, oats, wheat, and rice are susceptible to ZEN contamination [5,6]. Elevated levels of ZEN have been detected not only in raw feed ingredients such as wheat middlings, rapeseed meal, peanut meal, and fish meal but also in formulated animal feeds for pigs, poultry, and ruminants, thereby posing significant health risks to livestock [7]. Despite its varying stability in aquatic environments, ZEN may persist in nature and could threaten water sources such as lakes, rivers, and groundwater [8]. In addition, ZEN and its metabolites are among the most studied mycotoxin with endocrine disrupting activity, known to cause reproductive issues in people and animals [9]. ZEN was classified as a Group III carcinogen by the International Agency for Research on Cancer (IARC) in 1993 [10]. Moreover, ZEN is known to induce a range of long-term toxic effects, such as damage to reproductive, liver, immune, and genetic systems, and may also contribute to cancer development [10,11].

Pigs are widely recognized as the most vulnerable species to ZEN and its metabolites [11]. Absorption occurs through oral ingestion along with moldy food and feed [12]. Once absorbed, ZEN enters the bloodstream and is distributed in different organs, mainly the liver, the kidney, intestine, reproductive organs and adipose tissues [13]. It is metabolized by the liver [14] and gastrointestinal tract [15] via hydroxylation to form either toxic α- and β-Zearalenol (α- and β-ZEL), with α-ZEL being more toxic [16]. The resulting metabolites are then mainly eliminated through the kidneys [17]. The effect of ZEN on human and animal health has prompted some countries to establish appropriate, permissible levels in foodstuff intended for human and animal use. In the European Union, the maximum level for ZEN ranges between 0.5 and 400 μg/kg in various products intended for human and animal consumption [18,19]. The World Health Organization (WHO) identifies climate change, newly emerging infectious diseases, and human-induced disruptions as key global threats to public health [20]. In Italy, especially in the Campania region, recent years have seen notable changes in both the natural landscape and human activity [21]. Understanding how wildlife physiologically reacts to these environmental pressures is essential for successfully managing, tracking, and preserving wild animal populations. Wild boars can act as environmental bioindicators for ZEN pollution, due to their omnivorous diet and increasing presence in agricultural areas. Our studies conducted in Southern Italy have detected significant levels of ZEN and its metabolites in the liver of wild boars, suggesting that these animals are exposed to contaminated crops, particularly maize [22]. The liver, being the primary organ responsible for the detoxification and metabolism of this mycotoxin, is particularly susceptible to oxidative stress and inflammatory responses induced by ZEN, making it a critical target for assessing potential toxicological effects. This exposure not only reflects the environmental spread of mycotoxins but also raises concerns for human health, especially for those who consume wild boar meat. To our knowledge, there are no studies on the effects of dietary ZEN investigating its inflammatory effect in the liver of wild boars. Therefore, the objective of the present work was to evaluate the nitrosative stress and pro- and anti-inflammatory cytokines in the liver of wild boars positive for ZEN pollution in the Campania Region, Southern Italy.

## 2. Results

### 2.1. Evaluation of the Activity of the Pro-Inflammatory Cytokines in Liver Tissue of Wild Boars Positive for ZEN Contamination

**Figure 1:**
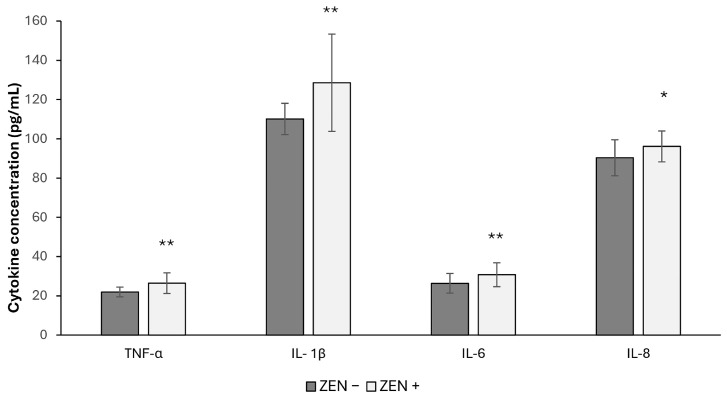
Effects of Zearalenone (ZEN) on Tumor necrosis factor-α (TNF-α), interleukin-1β (IL-1β), interleukin-6 (IL-6) and interleukin-8 (IL-8) levels in wild boars’ livers (*n* = 34 ZEN+; *n* = 14 ZEN−). Zearalenone negative group (ZEN−); Zearalenone-positive group (ZEN+). The results are expressed as the mean ± standard deviation (SD). * *p* < 0.05, ** *p* < 0.01 vs. ZEN−.

As can be observed in Figure 1, levels of pro-inflammatory cytokines in the hepatic tissues were significantly increased in the Zearalenone-positive samples (ZEN+) compared to the ZEN-uncontaminated ones (ZEN−). This up-regulation of cytokines was observed for TNF-α, with an increase of 20.41%, (** *p* < 0.01; t = 3.014); IL-1β: 16.75% (** *p* < 0.01; U = 114); IL-6: 20.32% (** *p* < 0.01; t = 2.862) and IL-8: 6.39% (* *p* < 0.05; t = 2.167).

### 2.2. Effect of ZEN on NF-κB (Nuclear Factor Kappa B) Pathway in Wild Boar Liver Tissue

**Figure 2:**
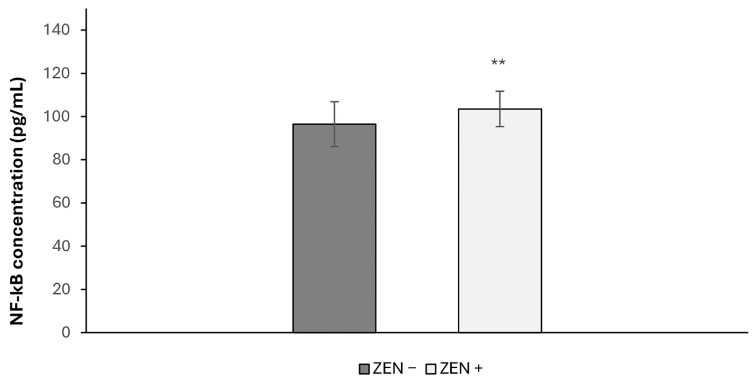
Effects of Zearalenone (ZEN) on NF-κB levels in wild boars’ livers (*n* = 34 ZEN+; *n* = 14 ZEN−). Zearalenone negative group (ZEN−); Zearalenone-positive group (ZEN+). The results are expressed as the mean ± standard deviation (SD). ** *p* < 0.01 vs. ZEN−.

The marker NF-κB is shown in Figure 2. The activity of NF-κB was increased significantly in the liver of wild boars belonging to the ZEN+ group compared with the ZEN− one. In fact, the NF-κB values increased by 7.30% (** *p* < 0.01; t = 3.42).

### 2.3. Evaluation of the Activity of the Anti-Inflammatory Cytokines, Interleukin-4 (IL-4) and Interleukin-10 (IL-10) in Liver Tissue of Wild Boars Contaminated with ZEN

**Figure 3:**
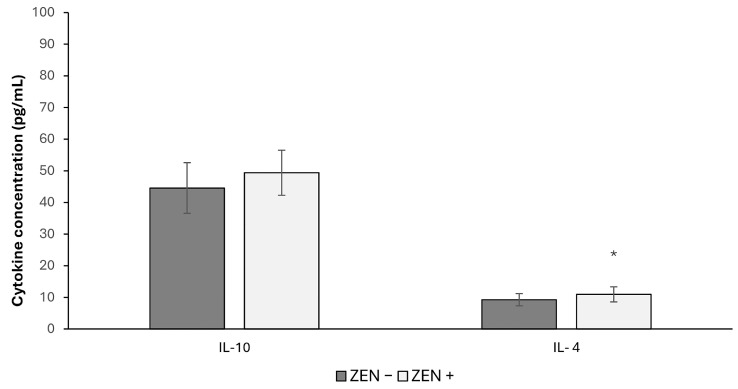
Effects of Zearalenone (ZEN) on interleukin-10 (IL-10) and interleukin-4 (IL-4) levels in wild boars’ livers (*n* = 34 ZEN+; *n* = 14 ZEN−). Zearalenone negative group (ZEN−); Zearalenone-positive group (ZEN+). The results are expressed as the mean ± standard deviation (SD). * *p* < 0.05, vs. ZEN−.

As shown in Figure 3, hepatic levels of interleukin-4 (IL-4) were significantly higher in ZEN+ than in ZEN− animals. In fact, an increase of 18.39% (* *p* < 0.05; U = 119) was observed. In contrast, a non-significant trend was observed in IL-10 (t = 1.95).

### 2.4. Effect of ZEN on Hepatic Tissue Levels of NO

**Figure 4:**
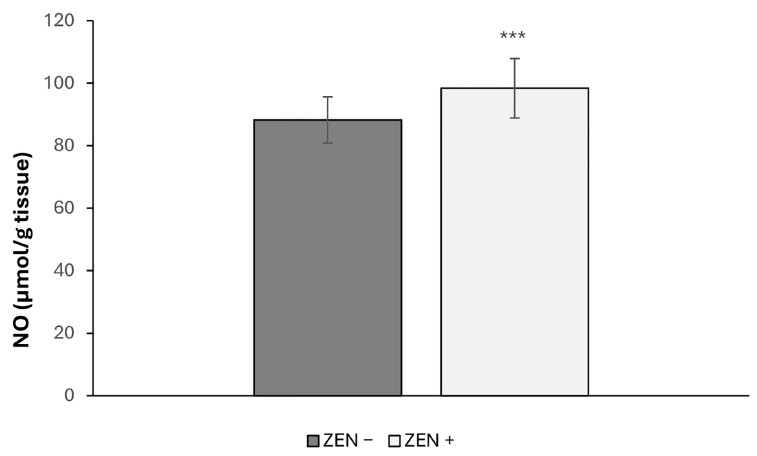
Effects of Zearalenone (ZEN) on NO levels in wild boars’ livers (*n* = 34 ZEN+; *n* = 14 ZEN−). Zearalenone negative group (ZEN−); Zearalenone-positive group (ZEN+). The results are expressed as the mean ± standard deviation (SD). *** *p* < 0.001, vs. ZEN−.

Liver nitrite levels were significantly higher in the ZEN+ group compared to the ZEN− one (Figure 4). Specifically, NO-related values increased by 11.53% in the ZEN+ group, with a highly significant difference (*** *p* < 0.001; t = 3.351).

### 2.5. Effect of ZEN on Hepatic Tissue Levels of iNOS

**Figure 5:**
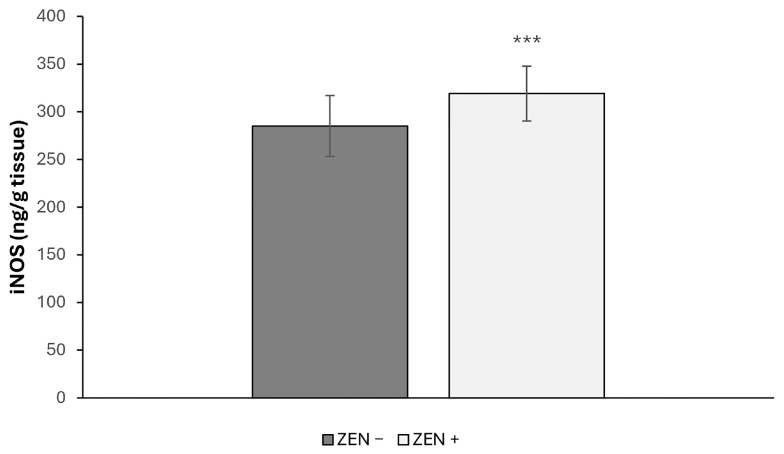
Effects of Zearalenone (ZEN) on iNOS expression in wild boars’ livers (*n* = 34 ZEN+; *n* = 14 ZEN−). Zearalenone-negative group (ZEN−); Zearalenone-positive group (ZEN+). The results are expressed as the mean ± standard deviation (SD). *** *p* < 0.001, vs. ZEN−.

Figure 5 shows the hepatic iNOS activity that was significantly raised in ZEN + group in comparison with the ZEN− group. In fact, the iNOS activity increased by 11.93% (*** *p* < 0.001; U = 75) when compared with ZEN− group.

### 2.6. Immunohistochemical Findings of Liver

**Figure 6:**
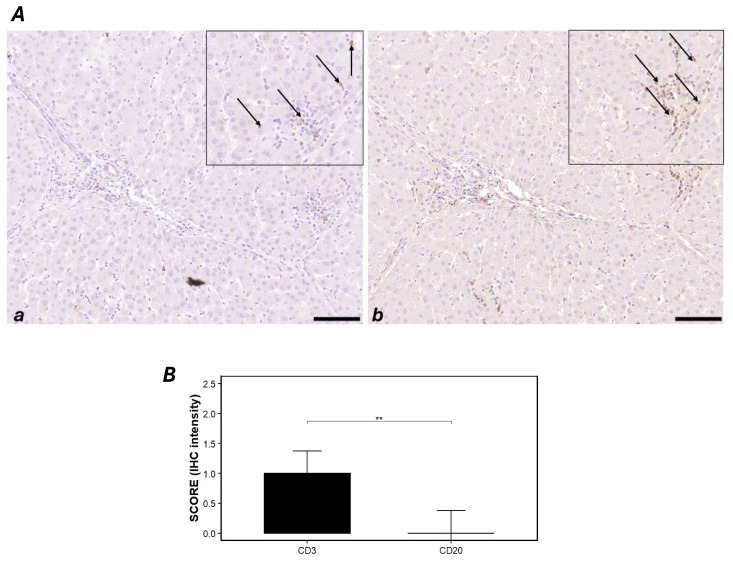
(**A**) **Immunohistochemical characterization of the inflammatory infiltrates in boar livers**. (**a**) Section of wild boar livers with chronic, multifocal, mild to moderate, inflammatory infiltrate, characterized by lymphocytes and rarer plasma cells; in the section, we used the antibody Anti-CD20, which showed a rare and mild positivity in the reaction (black arrows). (**b**) Section of wild boar livers with chronic, multifocal, mild to moderate, inflammatory infiltrate, characterized by lymphocytes and rarer plasma cells; in the section, we used the antibody Anti-CD3, which showed mild to moderate positivity in the reaction (black arrows). (**B**) **Statistical analysis.** Severity scores for immunohistochemical evaluation. Asterisks represent statistically significant differences between groups (** *p* < 0.01 CD3 vs. CD20; *p* = 0.00514). Original magnification, 200×, and high magnification, 400×. Scale bars, 20 µm.

The inflammatory infiltrate was chronic, multifocal, and mild to moderate, composed predominantly of small lymphocytes with occasional plasma cells. Immunohistochemically, the lymphocytic component showed diffuse membranous and cytoplasmic CD3 positivity, whereas CD20 expression was only rarely detected and, when present, was weak and scattered. Overall, these findings indicate a chronic, T-lymphocyte, predominant lymphoplasmacytic infiltrate. The global CD3 staining score was consistently higher than the CD20 score. CD3 staining was predominantly weak to moderate (scores 1–2) across most High-Power Fields (HPFs) and cases, whereas CD20 was negative to weak (scores 0–1) in the vast majority of fields (Table 1). These data support a T-cell–dominant inflammatory profile, with significantly higher IHC scores for CD3 than for CD20, consistent with a chronic T-lymphocyte-rich lymphoplasmacytic infiltrate (Figure 6). Positive and negative control immunohistochemical panels can be found in Figure S1.

## 3. Discussion

The wild boar has emerged as a valuable sentinel species not only for the surveillance of infectious diseases [23], but also for environmental monitoring, particularly in regions where agricultural practices and climatic conditions favor the proliferation of mycotoxigenic fungi. In Southern Italy, and especially in the Campania region, the expansion of wild boar populations has coincided with increased exposure to environmental contaminants, including *Fusarium*-derived mycotoxins such as ZEN [22]. Due to their omnivorous diet, wide-ranging foraging behavior, and ecological adaptability, wild boars are uniquely positioned to reflect the bioavailability of environmental toxins across diverse habitats. Their consumption of cereals, roots, and plant material often contaminated with mycotoxins makes them particularly susceptible to dietary exposure, thereby offering a natural model for assessing the ecological burden of ZEN contamination [22].

Our study conducted in the Campania region confirmed the presence of ZEN in the liver tissue of wild boars, suggesting widespread environmental contamination and underscoring the need for systematic surveillance [22]. The detection of ZEN in free-ranging wild boars not only reflects the contamination of local food chains but also supports the broader goals of the One Health framework, which emphasizes the interconnectedness of environmental, animal, and human health [24]. In this study, we evaluated hepatotoxicity in wild boars positive for ZEN exposure as the liver is essential to the body’s function, contributing to processes like digestion, metabolism, and immune defense, and serving as the primary site for the breakdown of mycotoxins. After ZEN ingestion, its concentration in the liver is second only to that found in the intestines [25,26,27], therefore the liver is exposed to a significant risk of toxin accumulation (EFSA 2011) [5]. Our previous study reported that ZEN induces oxidative stress-mediated hepatic damage [28]. This research provides compelling data confirming that ZEN induces substantial hepatic inflammation and oxidative damage in wild boars. The interplay between immune activation and nitrosative stress has been thoroughly investigated in toxin-induced inflammatory models and is regarded as a principal mechanism in the pathogenesis of chronic liver injury and other inflammatory conditions [29]. In this study, the significant increase in nitrite in the livers of ZEN+ samples compared to ZEN− ones implies increased formation of NO. As nitrite is a stable metabolic endpoint of NO, its increase serves as a reliable marker for greater NO bioavailability and subsequent nitrosative stress in the liver. This is further supported by the upregulation of iNOS, an enzyme typically activated during inflammatory responses. iNOS catalyzes the conversion of L-arginine to NO, which subsequently forms nitrite and nitrate as stable metabolites. The observed increase in hepatic iNOS in this study aligns with studies in the literature indicating that mycotoxins such as ZEN can stimulate NO production via iNOS induction, contributing to nitrosative stress and hepatocellular injury [30,31]. Our data also showed that the hepatic levels of NF-κB, TNF-α, IL-1β and IL-4 were significantly elevated in the ZEN+ group, whereas the increase in IL-10 levels was not statistically significant. NF-κB is a central transcription factor that regulates the expression of numerous pro-inflammatory genes, and its activation is a well-established consequence of oxidative stress and xenobiotic exposure [32]. Interestingly, the present study demonstrated that ZEN activated the NF-κB signaling pathway in wild boar liver cells, a pathway widely recognized for regulating the transcription of both anti-inflammatory cytokines, such as IL-10 and IL-4, and pro-inflammatory mediators including TNF-α, IL-1β, IL-6 and IL-8 [33,34,35]. TNF-α and IL-1β are key mediators of acute inflammation, initiating leukocyte activation and endothelial changes [36], while IL-6 and IL-8 contribute to leukocyte recruitment and tissue remodeling playing a dual role in both pro- and anti-inflammatory signaling [37]. Interestingly, the elevation of IL-4, typically associated with anti-inflammatory, may reflect a compensatory mechanism aimed at counterbalancing the pro-inflammatory milieu [38]. The cytokine profile observed here is consistent with previous findings in ZEN-exposed rodents and pigs, where NF-κB activation led to increased expression of inflammatory mediators and exacerbated tissue damage [39,40]. These data suggest that ZEN triggers a robust inflammatory cascade in wild boar liver, potentially impairing hepatic function and promoting chronic inflammation. Immunohistochemical analyses revealed a significant predominance of CD3-positive T lymphocytes and less CD20-positive B cells in the hepatic inflammatory infiltrate of ZEN-exposed wild boars. In fact, the livers from wild boars positive for ZEN exhibited a chronic, multifocal inflammatory infiltrate of mild to moderate intensity, composed predominantly of lymphocytes with occasional plasma cells. Immunohistochemistry demonstrated clear membranous/cytoplasmic positivity for CD3 and rarely weak staining for CD20, indicating that the lymphocytic component is dominated by T lymphocytes. These findings are consistent with low-grade, sustained hepatic injury in which an adaptive, T cell-mediated immune response predominates, directed against danger-associated molecular patterns (DAMPs) released by stressed hepatocytes or against neoantigens arising from ZEN-induced oxidative stress and metabolic alterations. This interpretation aligns with the literature recognizing ZEN as both hepatotoxic and immunomodulatory, capable of triggering pro-inflammatory responses and oxidative stress in the liver and other mucosal barriers [41]. Multiple studies have shown that ZEN interferes with key inflammatory and oxidative stress pathways (NF-κB, Nrf2/Keap1, MAPK), modulating the expression of cytokines (TNF-α, IL-1β, IL-6, IL-8) and downstream effector mediators [42]. Activation (or dysregulation) of NF-κB and an altered balance with Nrf2 have been implicated in increased susceptibility to hepatic injury and in the perpetuation of chemotactic signals for T cells [43]. In this context, the selective recruitment of T lymphocytes—documented here by CD3 positivity and CD20 negativity—is biologically plausible as the outcome of chronic, low-dose toxic stimulation [42]. This pattern most likely reflects chronic, low-dose exposure to ZEN in free-ranging animals rather than an acute inflammatory insult. The simultaneous increase in NF-κB activity, nitric oxide (NO) production, and the predominance of CD3+ T lymphocytes within the hepatic infiltrate support the interpretation of a chronic, low-grade inflammatory microenvironment, as opposed to a strong acute-phase response. In fact, NF-κB induction and iNOS, leading to NO overproduction, is a well-recognized mechanism linking low-grade inflammatory states with nitrosative stress and sustained hepatic injury [31].

Experimental models in rodents and pigs indicate that ZEN, even at relatively low doses, exerts hepatomorphological alterations, activates Kupffer cells, and modifies the perilobular connective framework. In parallel, ZEN can remodel both systemic and local immune landscapes, with effects that sometimes appear divergent (pro-inflammatory or immunosuppressive), likely depending on dose, exposure duration, co-exposure to other mycotoxins, and host physiological status. In our real-world setting (free-ranging wild boars), a chronic, mild-to-moderate, multifocal T-cell infiltrate is compatible with non-acute, ongoing exposure capable of sustaining a state of hepatic inflammation [44].

Overall, our results strengthen the concept that ZEN acts on the liver not only as a metabolic toxin, but also as an immune modulator compound, able to promote low-grade, T cell-enriched inflammatory microenvironments. From an eco-health perspective, this has One Health implications: wild boars, as environmental sentinels, may accumulate chronic mycotoxin exposures with consequences for hepatic health and immunocompetence; such subclinical conditions could influence susceptibility to hepatic pathogens and the outcomes of co-exposures [45]. The limited presence of adaptive immune cells may reflect either an early-phase response or immunosuppressive effects of ZEN on lymphocyte recruitment and function. These findings build on previously published morphological assessments of hepatic tissue in ZEN-exposed wild boars [22,28], providing a more detailed analysis of the immunophenotype of infiltrating cells. Taken together, the data presented here underscore the multifaceted impact of ZEN on hepatic physiology in wild boars, involving nitrosative stress, inflammatory signaling and immune cell infiltration. We would like to point out that the biological samples were obtained from wild boars hunted in their native ecosystems, a sampling context that offers a valuable ecological framework for assessing exposure to ZEN in wild populations. However, this approach inherently entails significant analytical constraints. Specifically, the quantification of oxidative stress and inflammatory biomarkers [45], presents substantial challenges in field-collected samples due to their inherent lability and sensitivity to environmental and temporal variables. These limitations compromise the reliability and representativeness of such measurements, particularly when sample collection and handling cannot be strictly controlled. To overcome these constraints, future research could benefit from the use of controlled experimental models, where post-mortem intervals and sample processing protocols are standardized. Alternatively, the development of field-adapted biobanking infrastructures coupled with targeted training programs for hunters and field personnel in proper sample preservation techniques may offer a pragmatic solution to enhance data quality and reproducibility in ecological and wildlife studies.

These results add to the expanding scientific understanding of the toxic impacts of Fusarium mycotoxins on wildlife, emphasizing the critical need for ongoing surveillance of environmental pollutants and their consequences for both animal well-being and ecosystem integrity.

## 4. Conclusions

This work provides evidence that Zearalenone (ZEN) exposure in wild boars induces a complex toxicological response involving oxidative stress, inflammatory activation, and immune cell infiltration in hepatic tissue. Overall, these findings reinforce the role of wild boars as sentinel species for environmental mycotoxin surveillance, particularly in Southern Italy where *Fusarium* contamination is prevalent and provides a comprehensive understanding of ZEN’s impact on wildlife health and supports the need for continued monitoring under the One Health framework.

## 5. Materials and Methods

### 5.1. Ethical Statement

Ethical approval was not necessary for this research, as the animals were lawfully hunted in their natural environment by licensed hunters, in accordance with the 2021–2022 annual hunting plan authorized by the Province of Avellino in the Campania region of Italy.

### 5.2. Sample Collection

A map of the Avellino province (Campania region, Southern Italy) indicating the hunting areas where wild boar (*Sus scrofa*) samples were collected has been published in previous study [22].

The sample set included 34 livers that tested positive for ZEN contamination using HPLC-FLD analysis in our previous research [28] and were categorized as the Zearalenone-positive group (ZEN+). Additionally, 14 livers of wild boar samples that tested negative for ZEN were classified into the Zearalenone-negative group (ZEN−). The study used a convenience sampling method for tissue collection. The analysis showed a mean Zearalenone (ZEN) concentration in the liver of 1.71 ± 0.339 ng/g (mean ± SE), which is well above the limit of detection (LOD) for liver tissue, established at 0.05 ng/g, as previously published by Longobardi et al. [22].

For subsequent biochemical analyses, a standardized procedure was followed. A 500 mg sample of liver tissue was collected from each wild boar. Each sample was thoroughly washed three times with phosphate-buffered saline (PBS) to remove blood cells and clots. The tissue was then immediately homogenized on ice using an electric tissue homogenizer (Tissue Lyser, Qiagen, Milano, Italy). After homogenization, the samples were centrifuged at 10,000 g for 10 min at 4 °C.

The resulting supernatants were collected and stored at −80 °C. This material was designated for future quantitative assessment of biomarkers related to nitrosative stress and pro- and anti-inflammatory cytokine levels. A separate aliquot of liver tissue was preserved in Bouin’s solution for subsequent immunohistochemical analysis.

### 5.3. Pro- and Anti-Inflammatory Cytokines Assay

Tumor necrosis factor-α (TNF-α, PTA00), interleukin-1β (IL-1β, ab100754), interleukin-6 (IL-6, P6000B), interleukin-8 (IL-8, P8000), interleukin-10 (IL-10, P1000), and interleukin-4 (IL-4, ab273205) levels in hepatic homogenate tissues were measured by enzyme-linked immunosorbent assay (ELISA) using specific kits (R&D Systems, Minneapolis, MN, USA and Abcam, Cambridge, UK) for porcine samples, following the manufacturer’s instructions [46]. Optical density (OD) was read at 450 nm using an ELISA plate reader (Glomax Multi Detection System, Promega, Milan, Italy). Concentrations were expressed as picograms of cytokine per milliliter of supernatant (pg/mL).

### 5.4. Nuclear Factor Kappa B (NF-κB) Activity Assay

NF-κB levels in hepatic homogenate tissue were measured using commercial ELISA kits (MBS702748, MyBioSource, Inc. San Diego, CA, USA) according to the manufacturer’s instructions. Absorbance was measured at 540 nm using a multi-well plate spectrophotometer (Glomax Multi Detection System, Promega, Milan, Italy). Concentrations were expressed as picograms of NF-κB per milliliter of supernatant (pg/mL).

### 5.5. Nitric Oxide (NO) Determination

NO levels in liver tissue were quantified using a spectrophotometric method with the Nitrite/Nitrate Assay Kit (MAK367, Sigma-Aldrich, Milan, Italy), following the manufacturer’s protocol. This method detects endogenous nitrite as a marker of NO synthesis. The procedure involves adding Griess reagent, which reacts with nitrite to form a purple azo dye. The absorbance of this compound was measured at 540 nm using a multi-well plate spectrophotometer (Glomax Multi Detection System, Promega, Milan, Italy). Results were reported in micromoles per gram of tissue (μmol/g tissue), and each sample was analyzed in triplicate.

### 5.6. Inducible Nitric Oxide Synthase (iNOS) Activity Assay

iNOS expression in liver tissue was assessed using a commercial ELISA kit (MAK532, Sigma-Aldrich, Milan, Italy), following the manufacturer’s protocol. This quantitative analysis used microplates pre-coated with a polyclonal antibody specific for iNOS (Sigma-Aldrich, Milan, Italy). Optical density values were measured using an ELISA plate reader (Glomax Multi Detection System, Promega, Milan, Italy). Results were reported in nanograms per gram of tissue (ng/g tissue), and each sample was analyzed in triplicate.

### 5.7. Histopathological Examinations

From the cohort of 34 wild boar liver cases described in the previous paper [28], we selected the 10 cases that showed the most significant histopathological lesions for immunohistochemical evaluation. From the paraffin-embedded blocks, serial sections of 3 μm were used for immunohistochemical analysis. To characterize the inflammatory infiltrate, we used antibodies against T lymphocytes (CD3) and B lymphocytes (CD20). Immunohistochemistry was performed using our established method [47,48,49]. Liver tissue sections were deparaffinized and rehydrated with a decreasing alcohol series, while endogenous peroxidase activity was blocked by incubation in 0.3% H_2_O_2_ in methanol (4:1 ratio) for 15 min. Antigen retrieval was performed by pre-treatment with microwave heating in a citrate buffer at pH 6.00. Pre-cooling, sections were washed with phosphate-buffered saline (PBS, pH 7.4, 0.01 M). Subsequently, immunohistochemistry was then carried out according to the manufacturer’s instructions for the MACH1 Universal HRP-Polymer Detection Kit (Code M1U539 G, L10, Bio-Optica, Milan, Italy). Primary antibodies included polyclonal rabbit anti-CD3 (Code ab5690, Abcam, Cambridge, UK) and polyclonal rabbit anti-CD20 (Bio-Optica, Milan, Italy), both diluted 1:100 in PBS and incubated overnight at 4 °C. Negative control tissues were treated with the same procedure, only the antibody was replaced with non-immune rabbit serum. For positive control tissues, multiple porcine lymph node sections were used to test both antibodies. For each case, ten 20× fields were randomly photographed with the Pannoramic II scan (3Dhistech, The Digital Pathology Company, Budapest Öv u. 3 1141, Budapest, Hungary). Immunohistochemical (IHC) positivity was scored semi-quantitatively, using a 4-tier ordinal scale across ≥10 non-overlapping HPFs for case (0 = no staining; 1 = weak, 1–10% immunopositive cells; 2 = moderate, 11–50% immunopositive cells; 3 = strong, 51–80% immunopositive cells) by two independent pathologists (E.D.N. and V.R.), blinded to group allocation, using light microscopy [47,48,49].

### 5.8. Statistical Analysis

Statistical analyses of pro- and anti-inflammatory cytokine activity levels and nitrosative stress levels in the livers of hunted wild boars positive for zearalenone contamination were performed using GraphPad (version 8.0; GraphPad Software Inc., San Diego, CA, USA). The Shapiro–Wilk and Kolmogorov–Smirnov tests were used to assess data distribution normality. If the data passed the normality test, means were compared using the Student *t*-test for independent samples. If the data did not meet the normality assumption, the nonparametric Mann–Whitney test was used (GraphPad Software 3.00, San Diego, CA, USA). A *p*-value < 0.05 was considered significant.

All histological analyses were performed in R (v4.x) within RStudio (Posit, Boston, MA, USA). Data were imported from an Excel worksheet and reshaped to a long format with two variables: marker (levels: CD3, CD20) and score (ordinal 0–2, representing the intensity of immunohistochemical staining). Because scores are ordinal and normality was not assumed, group comparisons were carried out using the Wilcoxon rank–sum test (Mann–Whitney U test, two-sided) implemented in the rstatix package. A *p*-value < 0.01 was considered statistically significant.

## Figures and Tables

**Table 1:** Liver. Severity scores for immunohistochemical evaluation.

LIVER	Score 0	Score 1	Score 2	Score 3
CD3	1	6	3	0
CD20	7	3	0	0

## Data Availability

The original contributions presented in this study are included in the article. Further inquiries can be directed to the corresponding authors.

## Supplementary Materials

The following supporting information can be downloaded at: https://www.mdpi.com/article/10.3390/toxins17110553/s1, Figure S1: Positive and negative control immunohistochemical panels.

## Abbreviations

The following abbreviations are used in this manuscript:

ZEN	Zearalenone
NO	Nitric Oxide
iNOS	Inducible Nitric Oxide Synthase
NF-κB	Nuclear factor kappa B
TNF-α	Tumor necrosis factor-α
IL-1β	Interleukin-1β
IL-6	Interleukin-6
IL-8	Interleukin-8
IL-10	Interleukin-10
IL-4	Interleukin-4
