# Supplementary material for: Evaluation of Activity of Pro- and Anti-Inflammatory Mediators and Nitrosative Stress in Liver Tissue of Wild Boars (Sus scrofa) Positive for Zearalenone (ZEN) Contamination in Campania Region, Southern Italy
Source: Toxins (Basel). 2025 Nov 5;17(11):553. doi: 10.3390/toxins17110553 (PMC12656457; doi:10.3390/toxins17110553)
Supplement: Supplementary file 1 [file toxins-17-00553-s001.zip › toxins-3950462-supplementary.pdf]

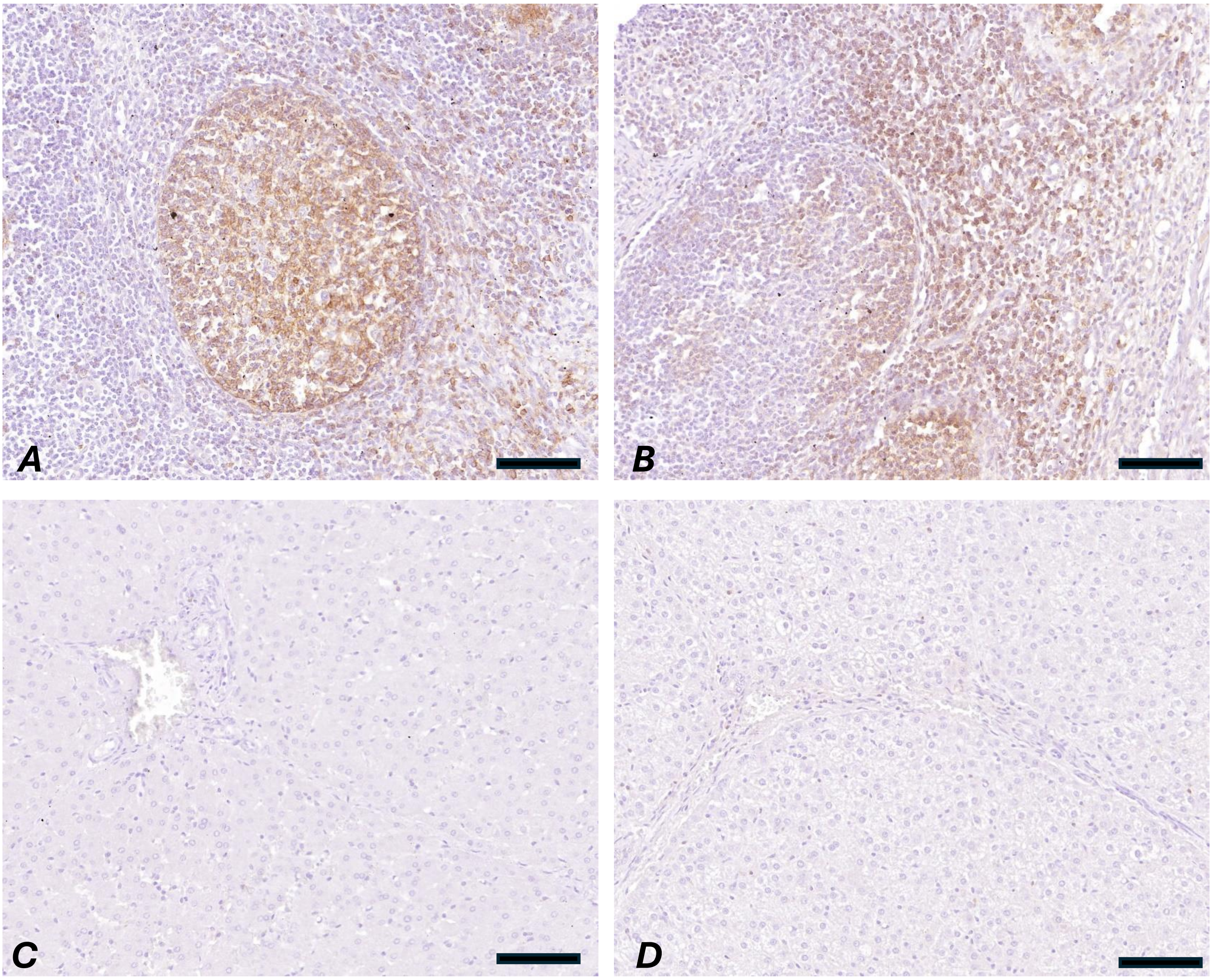

Figure S1: Positive and negative control immunohistochemical panels (A–D). (A) Positive control, CD20 on porcine lymph node section. **(B)** Positive control, CD3 on porcine lymph node section. **(C)** The group ZEN− didn't show positivity to the immunochemistry reaction for CD20. **(D)** The group ZEN− didn't show positivity to the immunochemistry reaction for CD3. Original magnification, 400×. Scale bars, 20 μm.
